# Is the efficacy of psychopharmacological drugs comparable to the efficacy of general medicine medication?

**DOI:** 10.1186/1741-7015-10-17

**Published:** 2012-02-15

**Authors:** Florian Seemüller, Hans-Jürgen Möller, Sandra Dittmann, Richard Musil

**Affiliations:** 1 Ludwig-Maximilian-University of Munich, Department of Psychiatry and Psychotherapy, Nussbaumstr.7, 80336 Munich, Germany

**Keywords:** effect size, general medicine, psychiatry, meta-analysis

## Abstract

There is an ongoing debate concerning the risk benefit ratio of psychopharmacologic compounds. With respect to the benefit, recent reports and meta-analyses note only small effect sizes with comparably high placebo response rates in the psychiatric field. These reports together with others lead to a wider, general critique on psychotropic drugs in the scientific community and in the lay press. In a recently published article, Leucht and his colleagues compare the efficacy of psychotropic drugs with the efficacy of common general medicine drugs in different indications according to results from reviewed meta-analyses. The authors conclude that, overall, the psychiatric drugs were generally not less effective than most other medical drugs. This article will highlight some of the results of this systematic review and discuss the limitations and the impact of this important approach on the above mentioned debate.

## Background

Psychiatry, psychiatric patients and psychiatric medication are still highly stigmatized. Psychiatry itself often promotes stigmatisation via close collaborations with the pharmaceutical industry and the disregard of psychotherapy, often overemphasizing the impact of psychopharmacology. A different line of criticism arises from recent meta-analyses questioning the efficacy of psychopharmacologic compounds. Examples are meta-analyses that have questioned the effectiveness [1] and safety of antidepressants [2]. With respect to the efficacy, the presumably high placebo effect in psychiatry has also often led to criticisms regarding the prescription of psychiatric drugs. These, together with other studies, have fuelled an overcritical attitude towards psychiatry and an ongoing debate about the use of psychiatric medications [3].

The British Journal of Psychiatry recently published a systematic review, planned and conducted by a psychiatrist who is an experienced member of the Cochrane collaboration and familiar with the pitfalls of meta-analyses - this article is a milestone in destigmatizing psychiatry and its pharmacological treatments [4].

## A new approach

In the review by Stefan Leucht *et al*., the authors compared the efficacy of psychiatric medications with general medicine medication by comparing effect sizes of meta-analyses [4]. For that purpose a total of 94 meta-analyses have been reviewed, of which 48 cover general medical medications in 20 medical diseases, and 16 meta-analyses cover psychopharmacologic compounds for 8 psychiatric disorders. Mean differences and standardized mean differences, as well as absolute and relative risk reductions, were calculated and the observational time of the respective trials was shown. While there were some medical drugs with impressive high effect sizes, overall it can be concluded that psychiatric drugs generally were not less effective than most other medical drugs [4].

For example, in patients with hepatitis C infection, the number of patients with no detectable virus at treatment end was increased from 1% to 38%, which corresponds to a standardized mean difference (SMD) of 2.27. Other impressive examples are proton pump inhibitors for the indication of reflux oesophagitis showing an increase of remitters from 28.3% with the placebo up to 83.2% (SMD: 1.39).

On the other hand, there are well established, but less effective first-line, options such as aspirin for the secondary prevention of cardiovascular diseases resulting in 8.2% participants per year with cardiovascular events taking placebo as compared to 6.7% with aspirin (SMD: 0.12). Statins for the same indication are somewhat in between those two ends. They reduce the event rate from 17.8% with placebo to 14.1% (SMD: 0.15). Other drugs for multifactorial diseases, such as, for example, metformin for the indication of diabetes, show a mortality rate of 14.6% against 21.7% with placebo (SMD: 0.27), and a mean fasting blood glucose level reduction of 1.84 mmol/l (SMD: 0.87). The use of angiotensin converting enzyme inhibitors in primary blood pressure reduces the cardiovascular events and mortality from 18.1% to 14.1% (SMD: 0.11).

These data are contrasted by psychiatric medications, such as, for example, lithium in relapse prevention for bipolar disorder resulting in 81.4% relapses with placebo and 36.2% with lithium maintenance treatment (SMD: 1.2). A substantial reduction of relapse rates can also be found in schizophrenia patients with a reduction from 57% relapse with placebo to 22% with an antipsychotic (SMD: 0.92). Less pronounced are effects that can be seen with cholinesterase inhibitors for dementia with 16.8% of patients without a cognitive decline taking placebo compared to 24.4% with an active drug (SMD: 0.26). In between are, for example, second generation antipsychotics or haloperidol for the indication of acute mania in bipolar disorder increasing the response rate from 30.8% with placebo up to 49.9% with an active drug (SMD: 0.44). Overall, sample sizes were smaller in psychiatric indications but still sufficient due to the robust effect sizes. In general, larger sample sizes were necessary in medical indications in order to find significant differences for smaller effects.

## Pitfalls and limitations of this approach

However, there are numerous limitations for this approach, (most of which are openly discussed by the authors themselves), which should be mentioned. For example, the translation of dichotomous variables into SMDs is only a rough estimate, which on the other hand was necessary in order to get a comparable measure across the diverse outcomes. A second important limitation is that different outcomes, such as the reduction of psychopathological symptoms assessed with rating scales and mortality outcomes are not easily comparable. However, untreated psychiatric disorders not only can lead to suicide but also may lead to negligence of, for example, severe comorbid somatic conditions, which also implies considerable health risks. In other words, psychopharmacologic compounds may also have effects which, according to Stefan Leucht, "could accumulate over time".

Another limitation of the study is the selection of the reviewed medical conditions. It was consensus based and not complete and this selection might have been biased. When reviewing meta-analyses, the publication bias must also be considered and side effects, as well as psychotherapy, should be considered. Furthermore, efficacy trials rarely allow conclusions regarding the effectiveness in day-to-day practice. Thus, as the authors truly acknowledge, this review is observational and qualitative by nature. Despite these limitations, this work raises some interesting and important implications and conclusions for both fields.

## What can we learn?

From a naïve point of view one might have expected that in psychiatry, since it covers "psychic disorders", the placebo effect (with its psychological component) might be higher and, as a consequence, the resulting true "pharmacologic" effect would be lower than in general medicine. One could also argue that in general medicine the pathophysiology and the mechanism of action of an active compound are somewhat clearer than in psychiatry and, therefore, the efficacy should be higher. But obviously neither seems to be universally valid. The astonishing similar efficacy of psychopharmacological medications and general medicine medications reminds us that many general medical conditions are, just like psychiatric conditions, of a "multifactorial" nature.

Therefore, it is not a surprise that in many medical and psychiatric conditions placebo response rates are relatively high. This may be especially true for idiopathic and functional medical conditions such as migraine, neurological disorders (such as Parkinson's disease), autoimmune disorders and asthma [5]. In other words, not only in psychiatry but also in a variety of common medical conditions, the placebo effect seems to be modulated by the fact that the intake of any drug is inevitably embedded in a specific psychosocial context which gives rise to distinct expectations. This raises the question of why this context may be widely accepted and common sense in medical conditions on the one hand, but leads to criticism with respect to the use of medications in psychiatry on the other. Amongst others, one important reason is that there are different areas of stigmatization in psychiatry. Stigmas concerning psychiatric illnesses can be separated and differ from stigmas concerning psychopharmacology [6].

In a survey on 1,088 healthy subjects in Germany from 1995, 70% of all respondents answered that medications for cardiovascular conditions are effective, while only 18% believed the medication might be effective in psychiatric conditions [7]. In the same survey, more than 40% of the respondents feared a loss of control with psychiatric medications, but only 10% feared the same when taking medication for cardiovascular conditions [7]. Moreover, 73% felt that high blood pressure should be treated in the first place with medications, but only 1% believed in the efficacy of psychotherapy in that indication. In contrast, severe paranoia should be treated with medication according to only 4% of the respondents, but 64% believed that psychotherapy would be a successful treatment [7]. Against this background, the review by Stefan Leucht *et al. *is an important contribution to overcoming the stigma against psychiatric medication and in putting its efficacy into a wider perspective.

On the other hand, it should be kept in mind that the paper by Stefan Leucht *et al. *only reviews pharmacological interventions which cover a part of all available medical treatment options. In psychiatry, for example, next to psychopharmacology and psychotherapy, there are other highly effective somatic treatments available, such as electro convulsion therapy (ECT) and transcranial magnetic stimulation (TMS). In general medicine, there are, of course, more and more minimally-invasive interventions available complementing the pharmacologic treatments, such as percutane transluminal coronary angioplasty (PTCA).

## Conclusions

This review is remarkable in two respects. Firstly, it gives the clinician an overview over effectsizes of diverse pharmacological treatments for medical and psychiatric conditions Secondly the effectsizes of psychiatric and medical conditions are compared with each other. Within its field it allows the clinician to align his clinical experience with the efficacy of treatments in day-to-day practice with the results from meta-analyses. In comparison to a second and different medical field, it shows us the limitations of pharmacological interventions in a holistic way. It thus, also, calls for more integrating research across the field borders. A good example of this is major depression, which is an independent risk factor for coronary artery disease [8], and myocardial infarction, in turn, is a known risk factor for major depression with a great impact on the six-month outcome [9]. To enlarge our treatment options and improve efficacy, we should continue to expand research across disciplines.

## Abbreviations

ECT: Electro Convulsion Therapy; PTCA: Percutane Transluminal Coronary Angioplasty; SMD: standardized mean difference; TMS: Transcranial Magnetic Stimulation

## Competing interests

FS received grants and research support from Lilly, Astra Zeneca and Glaxo-Smith Kline. He received honoraria from Lundbeck, Bristol-Meyers Squibb, Lilly and Astra Zeneca. HJM has received grants/research support from Lundbeck, Pfizer, Sanofi Aventis and Servier. He received Honoraria from Servier, Pfizer, Lilly, Jansen, Astra-Zeneca, Wyeth, Lundbeck and Sanofi Aventis. Prof Möller is a speaker or a member of advisory boards for Servier, Pfizer, Lilly, Jansen, Astra-Zeneca, Lundbeck, Sanofi Aventis and Wyeth. RM has received research support from AstraZeneca and Janssen-Cilag. SD declares that she has no competing interest.

## Authors' contributions

FS wrote all manuscript drafts. RM carried out the literature search and helped to draft the manuscript. HJM and SD read and critically revised all manuscript drafts. All authors read and approved the final manuscript.

## Authors' information

FS is specialized in the treatment of and research on affective disorders. FS and RM are consultants for psychiatry at the Department of Psychiatry at the Ludwig-Maximilians-University Munich. SD is senior psychologist at the Department of Psychiatry at the Ludwig-Maximilians-University Munich. HJM is the head of the Department of Psychiatry at the Ludwig-Maximilians-University Munich.

## Pre-publication history

The pre-publication history for this paper can be accessed here:

http://www.biomedcentral.com/1741-7015/10/17/prepub

## References

[B1] Kirsch I Deacon BJ Huedo-Medina TB Scoboria A Moore TJ Johnson BT Initial severity and antidepressant benefits: a meta-analysis of data submitted to the Food and Drug Administration PLoSMed 2008 5 e45 10.1371/journal.pmed.0050045 PMC2253608 18303940

[B2] Stone M Laughren T Jones ML Levenson M Holland PC Hughes A Hammad TA Temple R Rochester G Risk of suicidality in clinical trials of antidepressants in adults: analysis of proprietary data submitted to US Food and Drug Administration BMJ 2009 339 b2880 10.1136/bmj.b2880 19671933 PMC2725270

[B3] Greenberg G Manufacturing Depression: the Secret History of a Modern Disease 2010 New York, NY: Simon & Schuster

[B4] Leucht S Hierl S Kissling W Dold M Davis JM Putting the efficacy of psychiatric and general medicine medication into perspective: review of meta-analyses Br J Psychiatry 2012 200 97 106 10.1192/bjp.bp.111.096594 22297588

[B5] Moerman DE Meaningful placebos-controlling the uncontrollable N Engl J Med 2011 365 171 172 10.1056/NEJMe1104010 21751911

[B6] Castaldelli-Maia JM Scomparini LB Andrade AG Bhugra D de Toledo Ferraz Alves TC D'Elia G Perceptions of and attitudes toward antidepressants: stigma attached to their use-a review J Nerv Ment Dis 2011 199 866 871 10.1097/NMD.0b013e3182388950 22048139

[B7] Benkert O Kepplinger HM Sobota K Psychopharmaka im Widerstreit. Eine Studie zur Akzeptanz von Psychopharmaka und zur Darstellung in den Medien 1995 Berlin/Heidelberg: Springer

[B8] Lett HS Blumenthal JA Babyak MA Sherwood A Strauman T Robins C Newman MF Depression as a risk factor for coronary artery disease: evidence, mechanisms, and treatment Psychosom Med 2004 66 305 315 10.1097/01.psy.0000126207.43307.c0 15184688

[B9] Frasure-Smith N Lesperance F Talajic M Depression following myocardial infarction. Impact on 6-month survival JAMA 1993 270 1819 1825 10.1001/jama.1993.03510150053029 8411525

